# Editorial: Community series in novel insights into immunotherapy targeting tumor microenvironment in ovarian cancer: volume II

**DOI:** 10.3389/fimmu.2023.1338119

**Published:** 2023-11-29

**Authors:** An Tong, Hai-tao Wang, Xia-wei Wei, Xiao Liang

**Affiliations:** ^1^ Department of Gynecology and Obstetrics, Key Laboratory of Obstetrics & Gynecologic and Pediatric Diseases and Birth Defects of Ministry of Education, Development and Related Diseases of Women and Children Key Laboratory of Sichuan Province, West China Second University Hospital, Sichuan University, ChengDu, Sichuan, China; ^2^ Thoracic Surgery Branch, Center for Cancer Research, National Cancer Institute (NCI), Bethesda, MD, United States; ^3^ Laboratory of Aging Research and Cancer Drug Target, State Key Laboratory of Biotherapy and Cancer Center, National Clinical Research Center for Geriatrics, West China Hospital, Sichuan University, Chengdu, Sichuan, China

**Keywords:** ovarian cancer, tumor immune microenvironment, immunotherapy, cell therapy, bioinformatics analysis

Ovarian cancer (OC) is a highly lethal disease, which is often diagnosed at advanced stages (1). Although numerous articles have bit by bit complemented our understanding of tumorigenesis, effective therapeutic options for ovarian cancer are still lacking. In the last decade, notable successes in prolonging survival have been achieved by immunotherapy which encompass a spectrum of approaches, including immune checkpoint inhibitors (ICIs), Chimeric Antigen Receptor-T (CAR-T) cell therapy, vaccine therapy, and non-specific immune stimulants which aim to stimulate the immune system to attack cancer cells. However, the therapeutic response of immunotherapies in ovarian cancer remains suboptimal (2). Numerous studies suggest that this inadequacy might be attributed to the fact that ovarian cancer is a “cold tumor”, which is characterized by an immunosuppressive tumor microenvironment or a lack of tumor antigens (3). Therefore, it is necessary to find possible therapeutic approaches to activate the anti-tumor immunity in OC. In this Research Topic, we gathered eight articles to provide us with more comprehensive knowledge of the cancer–immune interaction parameters and application of immunotherapies in ovarian cancer.

With several antibodies, including anti-PD1 (programmed cell death 1), anti-PDL1 (PD1 ligand 1), anti-TIM3 (T-cell immunoglobulin mucin-3), and anti-CTLA4 (cytotoxic T lymphocyte-associated antigen-4) antibodies, approved for clinical anti-tumor treatment, ICIs have achieved the most promising success in recent years. However, the response rate of ICIs as a single treatment is relatively low in recurrent ovarian cancer (only 10-35%) (2). Therefore, to find possible combinations of therapeutic approaches for ovarian cancer patients, Zhang et al. reviewed a total of 141 articles in the field and highlighted the combination therapies which could benefit the immunotherapeutic response of PD-1/PD-L1 strategies in recurrent ovarian cancer. The authors suggested that prior combination therapies could induce immune cell infiltration which may convert the Tumor Micro-Environment (TME) from cold to hot and result in enhanced anti-tumor immunity in ovarian cancer.

Dendritic cells (DC) are an essential cell population in the tumor microenvironment. DC vaccination has been found to be a very promising approach to improve cancer immunotherapy efficacy and with little toxicity (4). Recent studies discovered that two conventional DC (cDC) subsets in the peripheral blood could be used as an alternative to monocyte-derived DC to improve the anti-tumor T-cell response. Therefore, Mastelic-Gavillet et al. characterized longitudinally phenotypic and functional properties of peripheral DC subsets among healthy donors and patients with ovarian cancers undergoing primary debulking surgery (PDS), interval debulking surgery (IDS), or at relapse. They discovered that compared to the PDS group, the IDS group had better preserved lymphocytes and cDC1s, which are fundamental for CD8 T-cell activation. By further analyses, they demonstrated that chemotherapy caused cDC1 depletion and impaired TLR3 responsiveness. Thus, they emphasized the importance of collecting cDC1 before chemotherapy which can be used as vaccines and combined with TLR3-targeted therapy.

Tumor-associated macrophages (TAMs) are another essential cell population in the tumor microenvironment. As TAMs can differentiate to anti-tumor M1 macrophages or immunosuppressive M2 macrophages, many macrophage-based anti-tumor strategies have been established (5). Among them, the blockade of the CD47/SIRPα pathway to restore the anti-tumor phagocytic capacity has emerged as a novel strategy. To gain a better understanding of the anti-tumor phagocytic capacity of TAMs in High-Grade Serous Ovarian Carcinoma (HGSOC), Brauneck et al. collected macrophages from the malignant ascites and peripheral blood from HGSOC patients and healthy controls. The authors discovered that the co-regulatory receptors were more frequently expressed on the TAMs isolated from HGSOC TME. As the blockade of T-cell Immunoglobulin and Immunoreceptor Tyrosine-based inhibitory motif domain (TIGIT) can significantly reduce the frequency of M2 macrophages, the authors combined the anti-TIGIT and anti-CD47 therapies in HGSOC patients leading to enhanced phagocytosis of TAMs.

In addition to targeting tumor microenvironments, cell-based therapies, particularly adoptive T-cell therapy, are alternative immunotherapy strategies for ovarian cancer. Wu et al. reviewed the adoptive T-cell therapies (ATC), especially T-cell receptor transduced-T cell (TCR-T) therapy applied in early phase clinical trials of ovarian cancer. Nevertheless, there are few well-documented markers and targets for TCR therapy in ovarian cancer. The authors believed that with the reducing cost of the next generation sequencing, the discovery for targeted tumor neoantigens will be cheaper and faster which will make the TCR-T a future personalized therapy. In this Research Topic, Yeku et al. presented another piece of research about bispecific T-cell engager antibodies (BiTEDs). Unlike CAR-T and TCR-T, BiTEDs redirect T-cell function independently of TCR recognition and are capable of serial cytotoxicity and secretion of inflammatory cytokines. They designed a BiTEDs against the MUC16 antigen (CA-125), which is universal in HGSOC, and discovered that it had significant anti-tumor effects in ovarian cancer, as well as combined with anti-Vascular Endothelial Growth Factor (VEGF) and anti-PD1 therapy. This study demonstrates the feasibility of BiTEDs treatment and provides preclinical evidence for immunotherapy.

Over and above the effective immunotherapeutic strategies, how to use standardized methods to measure the individual immunity parameters is critical. There are several articles in this Research Topic aiming to solve this problem. Wang et al. combined bioinformatics analysis and *in vitro* experiments to build a novel tumor mutational burden-based risk model that could predict prognosis, evaluate immune infiltration, and discover new therapeutic regimens in ovarian cancer. On the other hand, Ye et al. established a risk prediction model based on the RNA modification writer-related lncRNAs and evaluated its efficacy for serous ovarian cancer in prognostic and immune response prediction. Finally, there was another risk model based on differentially expressed necroptosis-related genes contributed by Wang et al. that could effectively predict the prognosis of ovarian cancer patients and explore the tumor microenvironment status.

In conclusion, with the emerging and burgeoning tumor genomic profiling and immune profiling, more and more promising and novel strategies will be provided for clinical diagnosis, individualized treatment, and immunotherapy in ovarian cancer.

## Author contributions

AT: Writing – original draft. H-TW: Supervision, Writing – review & editing. X-WW: Supervision, Writing – review & editing. XL: Supervision, Writing – original draft, Writing – review & editing.
